# Analyzing the use of fact-checking tools in disaster-risk reduction in Europe through the lens of the heuristic-systematic model

**DOI:** 10.1038/s41598-025-03099-0

**Published:** 2025-05-31

**Authors:** Nadejda Komendantova, Tahereh Zobeidi, Masoud Yazdanpanah

**Affiliations:** 1 Advancing Systems Analysis Program, International Institute for Applied Systems Analysis (IIASA), Laxenburg, Austria; 2 Department of Agricultural Extension and Education, Agricultural Sciences and Natural Resources University of Khuzestan, Mollasani, Iran

**Keywords:** Misinformation, Fake news, Fact-checking tools, Behavioral intention, HSM, Psychology, Human behaviour

## Abstract

Journalists, emergency responders, and the general public facing natural and anthropogenic disasters frequently disseminate emergency information via social media. The spread of fake news during disasters can, however, disrupt the crisis management process and increase victim numbers. Identifying false information can curb its spread and reduce its impact on people’s attitudes and behaviors. Understanding how and why people in a disaster situation use fact-checking tools is crucial, as disaster-risk messages containing false content can usually be detected using systematic or heuristic processing. This study applies the heuristic-systematic model (HSM) to analyze social media users’ intention to use fact-checking tools. The empirical study data derived from 202 questionnaires collected through an online survey of residents of countries of the European Union. The results of structural equation modeling show the credibility of using HSM to analyze the intention to use fact-checking tools. About 33% of the changes in people’s intention to use fact-checking tools are predicted by this model. This study has implications for the use of theoretical models in communication science to predict intention to use fact-checking tools in disaster risk-reduction situations.

## Introduction

Nowadays, methods of information exchange are changing due to the rapid spread of mobile technology and social media use for communication purposes^1^. Social media, largely defined as strategies and platforms that permit global operators to generate and spread data virtually, include social networking, micro-blogs, image- or video-sharing applications, and collaborative websites^2^. Such communication methods can act as channels for reporting timely disaster-related information to different populations^3–5^. Indeed, social media have various uses in disaster-risk reduction: releasing advance information for disaster preparedness; warning of impending adverse conditions; signaling disaster-related conditions to those at risk of, or affected by disaster; and, after a disaster event, linking public followers to recovery and response mechanisms^6,7^. In all such instances, social media are an effective means of conveying messages, increasing risk perception, and thus influencing behavioral decisions^8,9^. For the reasons just stated, journalists, emergency relief workers, and the public use social media during disasters as a tool for disseminating emergencies, time-sensitive, and first-hand information^2,5,7^. For instance, in Germany, some Facebook groups were set up to keep the public informed and to direct public volunteer fieldwork in response to floods^10^.

Social media have been used to manage many disasters: the earthquake and tsunami of 2011 in Japan; the ongoing COVID-19 epidemic; wildfires in 2019 in Moshav Mevo Modiim, Israel; flash floods in 2018 in the Aude region of France; industrial disasters in Venkatapuram (India) in 2020; and in terrorist attacks, for example, the Manchester Arena bombing. During the Boston Marathon bombings of 2013, Twitter developers created collaborative networks among the general public to broadcast warnings and alerts for potential threats, provide tips for minimizing damage, and help the population to recover from the bombings^5^. Compared with traditional media, social media have played a positive role in the exchange of information during the COVID-19 crisis, including the provision of health-related advice and psychological “first aid” with regard to risk perceptions, attitudes, experiences, and public insights into the disease^11,12^.

It is remarkable, however, that social media, to date, have been used in only 8% of disaster management cases^2^. This is because, despite their benefits, social media have also fueled the rapid spread of misinformation and rumor that can lead to panic and confusion^11,13^. Today, the concept of misinformation or “fake news” has become highly significant, particularly because incorrect social media messages can have potentially “dangerous” consequences. A single tweet by an “influencer”—be it head of state or celebrity—that presents incorrect information to potentially millions of viewers and readers can cause tremendous harm. This was seen in the promotion of hydroxychloroquine as a remedy for COVID-19 and the endorsement of UV light as potential treatments for the virus^14^.

In response to misinformation, a growing number of online facilities have been set up for the explicit recognition of false or misleading comments made online or through other agents. These online services can be separated into two classes: fact-checking services and verification services^15,16^. Nieminen and Sankari^17^ define fact-checking as a process for assessing the veracity of public claims, with the aim of discovering and disseminating whether a claim is true or not, by comparing it across different sources of information^18^. Fact*-*checking is a modern, identifiable category of journalism consisting of people or organizations that check for the accuracy and credibility of online content (e.g., FactCheck.org and StopFake)^19^. Fact-checking services usually include user groups that cover potentially wider ground than verification services, as they provide comprehensive analysis and evaluation of online claims or content that are potentially valuable to internet users^15^.

Verification services play a crucial role in confirming the authenticity of various online content forms, including text, images, and videos. These services employ algorithms to streamline and enhance the accuracy of the verification process. They are especially beneficial for professionals like journalists. For instance, TinEye, a tool accessible at http://tineye.com, scans the web for similar images, aiding journalists in scrutinizing online video sources^15,20^. Fact-checking represents a specialized application of verification, with verification services forming the foundational practice that facilitates fact-checking^16^.

Despite the importance of fact-checking, research shows that very few people use fact-checking tools^18^. A political study^21^ found that people have a negative view of fact-checking sites. Ejaz et al.^18^ point out that the scholarship on journalists’ use of fact checkers is still in its infancy. They proposed that although young journalists might be mostly unaware of or doubtful about such services initially, they would probably recognize their importance for journalistic analysis. However, they expressed hesitance to rely solely on these tools for verification.

Social media users are also undecided. Some users highlighted the practicality of such services, while others expressed strong distrust and skepticism towards them. Journalists, however, though unwilling to place blind trust in fact-checking services, acknowledged their potentially useful^22^. Lvidge and Steiner^23^ reported that when individuals first came across the concept of a fact-checker, they were unlikely to know what it was about (or to recognize its brand name). With repeated exposure to the product/service, they might develop a positive opinion about it and might intentionally visit fact-checking sites^21^. Various factors play a role in fact-checking including cognitive (awareness), emotional (attitudes), and behavioral aspects^21,23^. The majority of research points out how challenging it is to modify public opinion via fact-checking, and that people are probably not fully utilizing the available verification and fact-checking services. The motivations behind using such services are thus important to understand^15^.

Limited studies^24^ have been conducted on the use of fact-checking tools; however, none have been devoted to disaster risk-communication. To the best of our knowledge, there is no study that examines the intention to use such tools to fact-check emergency-related information. In other words, none of the studies have used an appropriate risk-communication framework to examine what factors influence people to fact-check in such circumstances. In this study, we use a theoretical framework based on the heuristic–systematic model (HSM) to focus on information-processing strategies used in risk-assessment communication^5,25^. According to the HSM, people process situations in two ways—via systematic processing and via heuristic processing. The systematic processing of information is qualitative and compares and contrasts with preceding knowledge; heuristic processing does not reflect all the pros and cons of the message, but emphasizes the simple cues in the message text^26^. The HSM posits that the dissemination of information precedes the formation of attitudes. As a process theory, it operates on the premise that attitudes develop as individuals are exposed to higher-quality information regarding pertinent subjects^27^. As such, it is particularly well suited for use in risk communication studies because it can effectively link questions about where people receive threat information from, how they engage with it, and how this provides insights into risks.

The HSM was developed to apply credible persuasion settings in which individuals’ primary motivation is to achieve attitudes consistent with the relevant facts^28^. This is why analyses for this paper are based on HSM theory.

The Heuristic-Systematic Model (HSM) was chosen as the theoretical framework for this study due to its focus on information-processing strategies in risk-assessment communication. As outlined in the paper, HSM categorizes individuals’ processing of information into two types: systematic and heuristic. The systematic processing is detailed and involves comparing new information with prior knowledge, while heuristic processing relies on simple cues in the message, without fully considering its pros and cons. These features make HSM particularly well-suited for risk communication studies, as it effectively links how people receive threat information, how they engage with it, and how it influences their attitudes.

Moreover, HSM was selected because it focuses on attitudes being shaped by the exposure to higher-quality information, a central aspect of our study. The model’s emphasis on credible persuasion, where individuals are motivated to align their attitudes with relevant facts^28^, directly relates to our examination of how information is processed in risk contexts.

We are also aware of certain critiques of HSM. One common limitation is that the model may be less effective in situations where individuals’ motivations are more complex than simply aligning attitudes with factual information. This study addresses this limitation by focusing on specific risk communication contexts and considering contextual factors that might reduce these shortcomings. For instance, through the use of textual data and empirical evidence related to how users process information in real-world scenarios, our study aims to minimize the theoretical limitations of HSM.Investigators have claimed that the HSM can be used with respect to a broader variety of rationality-seeking settings than other dual procedure theories like the elaboration likelihood model (ELM)^29^. Existing studies have applied HSM in various fields such as information technology, business and message management, health, and risk communication.

A review of studies shows that the theory is applicable in various fields, for example: the decision to retweet disaster topics^5^; the effectiveness of phishing attacks and the evaluation of the credibility of information by phishing victims^29^; anti-nuclear behavioral intentions^30^; risk perception after the Fukushima nuclear accidents^31^; Android data processing and service-access notification^32^; and consumer perception of online product reviews^33^. To the best of our knowledge, HSM has never been used to investigate the intention to use fact-checking tools, another area in which HSM can be useful, as disaster-risk messaging contains minimal false content that can usually be detected with sufficient systematic distribution. This study presents a theoretical contribution to the use of HSM regarding fact-checking tools.

Some studies in the field of fake information have focused on the intention to share fake information^34^, as there is a lack of literature on the practice and understanding of fact-checking by people in general. This study seeks to bridge this knowledge gap by examining the determinants of intention to use fact-checking tools in European countries facing multiple disasters. As far as we know, this is the first study to test cognitive processes focusing on the intention to fact-check. Based on previous studies, people who realize that the news they are hearing is based on untruths may show less intention to share the news with their social networks^35^. By identifying the factors affecting the use of fact-checking tools, this study can play a role in reducing the damage to communities that are victims of disasters.

## Background: heuristic-systematic model

The HSM is a model of information processing^27^ that evolved from the study of persuasion in social psychology, which investigates how conventional messages can modify recipients’ attitudes. The HSM’s essence is that people in the process of being persuaded first assess the trustworthiness of the “persuasion message” using a combination of heuristic and systematic processing; a multiplicity of factors have a bearing on the outcome of the assessment^29,31^. People’s perception of the information’s credibility can help them distinguish a valid message from a fake one.

Systematic processing carefully inspects the information contained in the message to assess its validity. Message viewers will make a significant cognitive effort to process information and actively evaluate the arguments and validity of statements made^36,37^. Heuristic processing, on the other hand, uses influences entrenched in or surrounding a message (heuristic cues), such as source, format, length, and topic, to quickly assess credibility^29^. In the heuristic mode, however, people make little effort to judge the credibility of messages and rely on easily available information such as the source’s identity or non-content cues to decide whether it is valid^34,38^. Todorov et al.^39^ noted that systematic information processing in the HSM suggests that people consider all relevant pieces of information, process them in detail, and make judgments based on them. Systematic processing, however, requires more time and cognitive resources. According to the HSM, people lacking motivation or ability tend to limit the time and cognitive resources they devote to heuristic processing^29^. The HSM posits that people use either one or both methods to make a judgment when under stress^40^. Systematic and heuristic policies are used in stand-alone or concurrent mode^30^. Disaster communication literature^5,25,41,42^ emphasizes the importance of heuristic processing information for emergency communication because it can minimize information processing and lead to a faster reaction to disaster.

Heuristic processing is usually compared with algorithms used in artificial intelligence or normative rules of utility maximization. These algorithms are clear and precise instructions that guarantee the correct result, but where cognitive resources are limited, they can be laborious and time-consuming and thus impractical^43^.

Three predictors—Information sufficiency, information seeking, and source credibility—could predict whether heuristic or systematic processing come into play^44^. The sufficiency principle of the HSM represents the notion that people pursue a balance between two goals: expending minimum energy and being confident about their own decisions. The link between motivation and information processing thus arises from this interaction between the principles of minimum effort and having enough information^45^.

The HSM assumes that individuals do not necessarily strive to evaluate the credibility of information to the point of maximum reliability or accuracy. When their evaluations are good enough, they stop processing. This idea is supported by a concept unique to the HSM called the adequacy threshold of “anticipated critical self-assurance” which people wish to achieve when making a choice under certain conditions^46^. The HSM recommends that when information sufficiency meets critical self-confidence, an individual feels confident enough to make a judgment^27^. People use heuristic processing when they feel they have enough information to feel confident about the decisions they will be making^45^; if they do not feel they have enough information, they will try systematic processing^30^. This study thus hypothesizes that the perception of information sufficiency is one of the predictors of whether heuristic and systematic information processing are used.

The HSM principle of the least effort postulates that individuals prefer expending less energy to making more effort, not because they are lazy, but because their processing is economical, using their own cognitive resources solely when they really need them—that is, when their own welfare is complicated. According to this principle, heuristic processing is “evasive,” necessitating much less energy and taking up much less space than systematic processing. While heuristic processing fully satisfies the belief in least effort, systematic processing classically leads to greater amounts of self-confidence and thus can improve the sufficiency principle^45^. However, information seeking affects both types of information processing. Johnson^44^ shows that searching for information has positive effects on systematic processing and negative effects on heuristic processing. In other words, information seeking may motivate people to process information systematically.

Communicators play an important role in delivering persuasive messages^45^, and HSM researchers have tried to classify what types of methods are needed to persuade people. Perceptions of source credibility significantly guide the way in which risk information is processed and can influence related behavior^47^; and the impact of source credibility or trust in the source on judgments related to risk is often discussed.

Source credibility refers to interpersonal trust associated with the presence or absence of certain source characteristics^48^. Source credibility has long been thought to include perceptions of a source’s trustworthiness and expertise^47^. Indicators of source credibility such as apparent competence and trustworthiness are positively related to both heuristic and systematic processes. This study thus hypothesizes that source credibility is one of the predictors of heuristic and systematic processing of information.

The outcomes of exploratory and systematic processing determine the validity of information. The credibility of the information or message relates to the degree of influence of different message characteristics on the perception of those assessing how credible a message is^49–51^. Study Metzger^52^ have confirmed the effect of systematic and heuristic processing on the evaluation and judgment of the credibility of information.

**Fig. 1:**
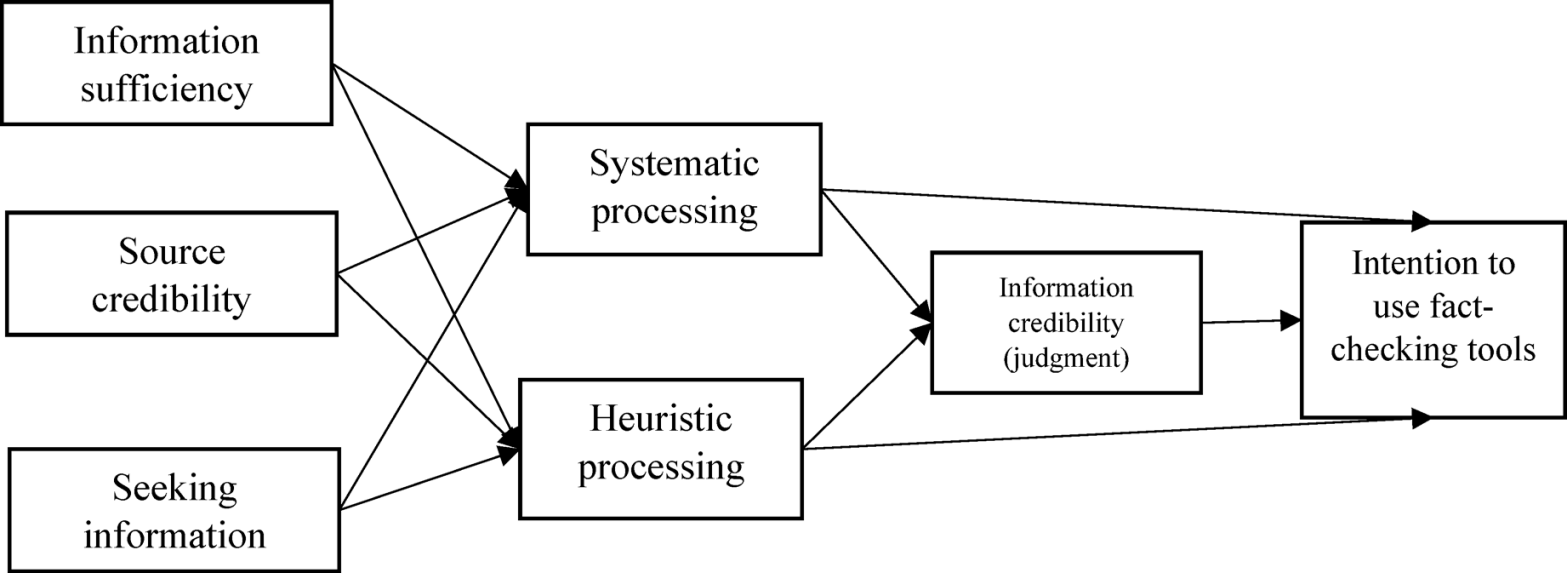
Conceptual model based on systematic-heuristic processing for fact-checking.

We have referred to the social psychology literature to explain the components and theoretical extensions of the model^33^. In early research on dual-process models such as Eagly and Chaiken work^45^, it was claimed that information processing influenced attitude formation/change or that attitudes influenced decisions. Given that previous research has established a generally close relationship between attitude and intention, this study emphasizes the influence of two process characteristics and examines the extent to which they directly influence intention^33^. For example, Xu et al.^9^ claimed that information credibility is important for real disaster communication and that its strength affects residents’ intention to evacuate during an earthquake. According to Zhang et al.^33^, the quality of the reasoning or systematic processing can influence consumers’ behavioral intention. In their study on HSM, Park et al.^53^ found that both systematic and heuristic processing influenced participants’ perceptions of product reviews and thus their purchase intentions. We therefore hypothesized that information credibility as well as heuristic and systematic processing affect the intention to use fact-finding tools. Figure 1 shows our research framework based on systematic–heuristic processing for carrying out reality checks.

## Research methodology

### Research design

To investigate the factors affecting the intention of social media users to use fact-checking tools, a study was conducted using an online questionnaire, which led to the compilation of 202 usable responses. Data collection was carried out in April and May 2022. The geographical area covered was EU countries, and the questionnaire language was English. Due to the geographical size and high population of European countries, the European Union was divided into sex categories based on geographical regions (https://www.infoplease.com/atlas/europe) consisting of Scandinavia, Western Europe, Southern Europe, Central Europe, Southeast Europe, and Eastern Europe. One country was then randomly selected from each category, including Sweden, United Kingdom (UK), Italy, Germany, Croatia, and Lithuania. Thirty questionnaires were selected from each country; a total of 210 questionnaires were collected but eight were removed due to incompleteness and a high number of missing values.

Two sampling approaches were chosen for data collection. An online version of the final questionnaire was prepared. We distributed the URL link of the questionnaire among potential social media users. We announced an invitation on LinkedIn, ResearchGate, and other social media. We also distributed the questionnaire to people through Facebook and WhatsApp groups. To reach more respondents, we shared the questionnaire in selected countries, using a list of email addresses.

Respondents were invited to complete the questionnaire if they met two criteria: (i) they were born in one of the six countries, and (ii) they used at least one of the three social media platforms—Instagram, Twitter, or Facebook—almost daily for at least 15 min to access news. If respondents reported not using any of these platforms, they were excluded from the survey.

### Measures

The seven constructs of the framework were measured using a total of 28 observed variables. These items were derived from a literature review, with some modifications made to adapt them to the context of the research^29–31,33,54^.

The items used for each construct along with the source are listed in Table 1. Those sampled were requested to specify their level of agreement with each of the items using a 5-point Likert scale including (Strongly disagree = 1, disagree = 2, 3 = Neither agree nor disagree, 4 = agree, 5 = Strongly agree). The assessment of the appropriateness of the items was conducted via a pilot study of 30 respondents who were all residents of European countries (without being separated into countries(; the investigations show that the items were easily understood by the respondents. Construct reliability or internal consistency show how well a construct is measured by indicators. Cronbach’s alpha criterion is used to evaluate construct reliability^55^. Construct reliability is good when Cronbach’s alpha values are 7.0 or higher. As Table 1 shows, all constructs have good internal correlation.

**Table 1:** Items, sources and their descriptive statistics.

Sources		Items	FL	t	Mean	sd
Zhu et al.^30^		Seeking information				
	SI1	I follow the latest news related to these disasters every day on social media.	0.80	10.18	3.69	0.76
	SI2	I have to search for more information about these disasters.	0.79	10.05	3.83	0.82
	SI3	I want to search for information about these disasters.	0.69	–	3.79	0.70
Zhu et al.^30^		Information sufficiency				
	IS1	If I have little experience of disaster, I often use social media.	0.53	–	3.72	0.81
	IS2	I would like to know more about the progress of disaster risk reduction.	0.73	6.69	3.31	0.70
	IS3	The information issued by government sectors is insufficient.	0.85	12.82	3.85	0.81
	IS4	I like to apply the social media when dealing with disasters.	0.82	12.71	3.49	0.84
Zhang et al.^33^ Ryu and Kim^31^ Luo et al.^29^		Source credibility				
	SC1	The social media providing the disaster information is trustworthy.	0.83	–	3.14	0.75
	SC2	The social media providing disaster information is reliable.	0.85	14.48	3.19	0.75
	SC3	Those who provide messages about risk disasters on social media have good knowledge of the topic (are experts on the topic).	0.86	15.29	3.03	0.74
Zhu et al.^30^ Ryu and Kim^31^ Luo et al.^29^		Systematic processing				
	S1	I think about the importance of information related to these disasters in my daily life.	0.60	–	3.46	0.90
	S2	I take a cautious stance toward seeking information on disaster risks	0.84	12.63	3.72	0.69
	S3	I tried to judge disaster risk issues based on objective data.	0.73	12.65	3.74	0.69
Ryu and Kim^31^ Luo et al.^29^		Heuristic processing				
	H1	I accepted all information about disasters presented by experts without hesitation.	0.60	–	3.29	0.83
	H2	We accept all news content about disasters from social media without any filtering.	0.64	6.99	2.95	0.87
	H3	I accept all the information on disasters presented by experts without seeking any more information.	0.81	7.47	3.05	0.84
Zhang et al.^33^ Hur et al.^54^		Information credibility				
	IC1	The messages provide violent information on disasters (reverse item)	0.63	–	4.03	0.57
	IC2	The messages provide incomplete and biased news on disasters (reverse item)	0.73	0.8.56	3.60	0.70
	IC3	The messages provide necessary information on disasters.	0.79	9.07	3.64	0.70
Researcher-developed		Intention to use fact-checking tools				
	I1	I would like to use fact-checking tools to check for social media misinformation on disasters.	0.67	–	3.79	0.76
	I2	I plan to use fact-checking tools to check for social media misinformation on disasters.	0.91	12.63	3.55	0.83
	I3	I intend to use fact-checking tools to check for social media misinformation on disasters.	0.97	12.66	3.66	0.80

### Data analysis

The data analysis was performed using AMOS (Version 24) software for linear structural equation modeling (SEM). SEM is a statistical technique that employs a confirmatory approach to investigate the structural theory of cause-and-effect relationships influencing various phenomena. It is widely adopted across multiple disciplines due to its comprehensive ability to quantify and assess underlying theories. SEM integrates confirmatory factor analysis (CFA), multiple regression analysis, and path analysis systematically, with estimation conducted using the Maximum Likelihood Estimation method and missing data addressed through data imputation.

SEM comprises two primary stages: the measurement model and the structural model. The measurement model assesses the relationships between indicators (items) and latent constructs, primarily relying on CFA. Meanwhile, the structural model illustrates the relationships among constructs within the model, evaluated through the determination of endogenous constructs, significance, and path coefficients.

To validate the measurement model, convergent validity and discriminant validity were assessed. Convergent validity ensures that items within a construct are closely associated due to theoretical relevance, evaluated through indices such as composite reliability (CR), average variance extracted (AVE), and factor loadings. CR assesses internal consistency, while AVE summarizes convergence. An AVE value higher than 0.5 and CR value higher than 0.7 are considered as acceptable thresholds. Factor loadings, indicating the correlation between observed variables and constructs, should minimally exceed 0.5. Discriminant validity confirms the extent to which a construct’s measurement is distinct from others. It is affirmed when the square roots of AVE for a construct exceed its correlations with other constructs.

We used modification indices to improve the model fit. Model fit assessment includes metrics such as the relative chi-square ratio (χ^2^/dof), goodness-of-fit index (GFI), comparative fit index (CFI), incremental fit index (IFI), normed fit index (NFI), Tucker-Lewis index (TLI), root mean square error of approximation (RMSEA), and standardized root mean square residual (SRMR). A model is considered well-fitting when the relative chi-square ratio is below 3. Additionally, GFI, CFI, IFI, NFI, and TLI values closer to 1 indicate better fit, with values above 0.95 considered very good and above 0.90 considered good. An RMSEA value below 0.08 and an SRMR value below 0.06 represent acceptable thresholds.

### Ethical approval and informed consent

All procedures performed in studies involving human participants were in accordance with the ethical standards of the institutional and/or national research committee and with the 1964 Helsinki declaration and its later amendments or comparable ethical standards. Informed consent was obtained from all subjects involved in the study. All materials and methods are performed in accordance with the instructions and regulations and this research has been approved by an ethical committee at International Institute for Applied Systems Analysis, Austria.

## Results

### The profiles of the participants

The participants were 48.5% (98) male and 51.5% (104) female. The youngest respondent was aged 18 and the oldest 57 (M = 34.88, SD = 9.61), education levels were 7.4% high school, 9.4% college, 1.0% associate degree, 19.3% Bachelor’s, 26.7% Master’s, 1.0% professional qualification, and 35.1% doctorate.

**Fig. 2:**
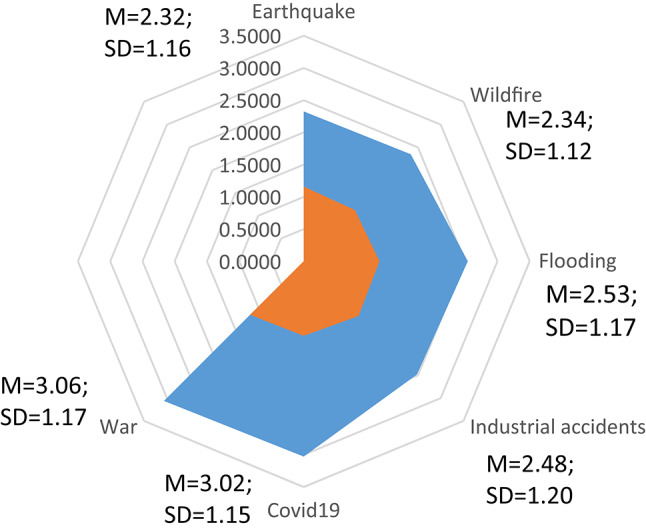
Using social media to obtain information.

The respondents were asked to indicate which of the three social media platforms they had used for (i) viewing and reading general news and (ii) reading news related to disasters. According to the answers, to obtain general news, 45.9% of respondents used Facebook, 39.2% used Instagram, and 14.9% used Twitter. For disaster-related news, 54.3% used Facebook, 28.7% used Instagram, and 17% used Twitter, showing that some of those who used Instagram for general information preferred to rely on other platforms for disaster news, on average, people spent 113 min a day on social media with an average of 32 min being devoted to reading information about disasters. As shown in Fig. 2, the responders for health disasters such as COVID-19 (M = 3.02) and (M = 3.06) receive more information from social media (Facebook, Instagram, Twitter) about war than about other disasters.

### Correlation between HSM constructs and intention to use fact-checking tool

The overall fit of the measurement model is very good. The relative chi-square value is 1.685, less than the suggested threshold of 3. The value of the RMSEA was 0.058, which also indicates an excellent model fit. In addition, SRMR = 0.0574, was less than the threshold value of 0.06, indicating a good fit of the model. The values of CFI = 0.961, NFI = 0.912, IFI = 0.962, and GFI = 0.905, and TLI = 0.939 were obtained, which are higher than 9.0 and indicate the excellent suitability of the model. As Table 2 shows, the acceptable thresholds of, factor loading, AVE and CR were higher than 0.7 and 0.5, and the square root of AVE was higher than the correlation between them. As a result, the research model has acceptable convergence and discriminant validity.

**Table 2:** Convergent and discriminant validity.

	Seeking information	Insufficient information	Source credibility	Systematic processing	Heuristic processing	Information judgment	Intention to use fact-checking tools
Seeking information	.76^a^						
Insufficient information	0.394**	.74^a^					
Source credibility	0.351**	0.103	.84^a^				
Systematic processing	0.398**	0.215**	0.267**	.73^a^			
Heuristic processing	0.463**	0.228**	0.266**	0.359**	.68^a^		
Information judgment (credibility)	0.591**	0.407**	0.142*	0.369**	0.372**	.71^a^	
Intention to use fact-checking tools	0.389**	0.163*	0.188**	0.327**	0.273**	0.157*	.85^a^
AVE	0.580	0.552	0.717	0.533	0.475	0.518	0.739
CR	0.805	0.827	0.884	0.771	0.727	0.762	0.893
Mean	3.77	3.59	3.12	3.09	3.64	3.76	3.67
SD	0.65	0.63	0.67	0.67	0.62	0.54	0.75

^a^Square root of AVE.

### Structural model

The overall fit of the structural model is very good. The relative chi-square value is 1.682, less than the suggested threshold of 3. The RMSEA value was 0.058 and SRMR was 0.716, which indicates excellent model fit. Moreover, CFI = 0.960, NFI = 0.910, IFI = 0.961, and GFI = 0.905 and TLI = 0.939 values were obtained, which are higher than 9.0 and indicate the excellent suitability of the model.

**Fig. 3:**
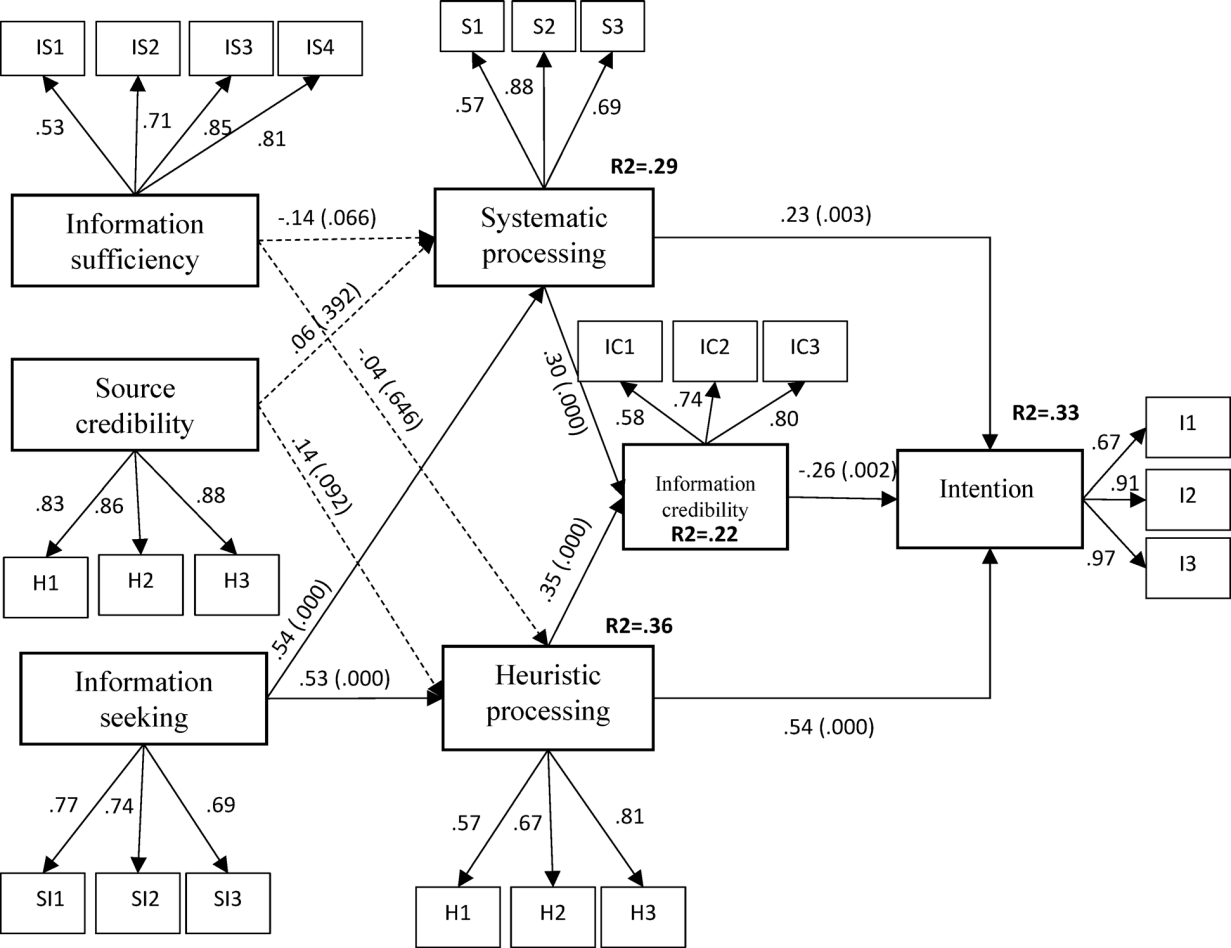
Structural equation modeling results.

Seeking information was the key determinant of heuristic processing (β = 0.53, t = 4.672) and systematic processing (β = 0.54, t = 4.793). Both systematic processing (β = 0.30, t = 3.385) and heuristic processing (β = 0.35, t = 3.633) have a significant effect on information judgment. The effect of systematic processing (β = 0.23, t = 2.948), heuristic processing (β = 0.54, t =− 2.741) and information credibility (β = -0.26, t =− 3.072) on intention to use fact-checking tools were also supported (Table 3). The research model showed 22% and 33%, respectively, of variance of credibility information and intention to use fact-checking tools (Fig. 3).

**Table 3:** Results of structural equation modeling.

Hypothesis			Unstandardized regression weights	SE	CR	*P*
Seeking information	→	Systematic processing	0.615	0.128	4.793	***
Seeking information	→	Heuristic processing	0.561	0.120	4.672	***
Insufficient information	→	Systematic processing	− 0.126	0.068	− 1.841	0.066
Insufficient information	→	Heuristic processing	− 0.03	0.065	− 0.460	0.646
Source credibility	→	Systematic processing	0.052	0.061	0.857	0.392
Source credibility	→	Heuristic processing	0.108	0.064	1.684	0.092
Systematic processing	→	Information credibility	0.177	0.052	3.385	***
Heuristic processing	→	Information credibility	0.225	0.062	3.633	***
Systematic processing	→	Intention to use fact-checking	0.297	0.101	2.948	0.003
Heuristic processing	→	Intention to use fact-checking	0.759	0.156	4.858	***
Information judgment	→	Intention to use fact-checking	− 0.557	0.138	− 3.072	0.002

## Discussion

Despite the seriousness of the costs and problems of fake news in social media and the importance of the social media in a disaster situation, there is still considerable uncertainty about the factors affecting the intention of social media users to use fact-checking tools to process information. Fake information can cause more damage to society during disasters than under normal conditions. In addition, as identifying fake information can prevent the spread of false information by users, using simplified fact-checking tools can help reduce disaster risk. This study thus aims to predict the factors affecting the intention to use fact-checking tools using HSM.

Although this study is neither a controlled experiment nor a comprehensive test of hypotheses, its design and results provide some support for the reasonableness of using HSM to study the intention to use fact-checking tools, as many hypotheses were confirmed and about 33% of people’s intention is predicted by this model.

Information-seeking has strongly influenced both systematic and heuristic processing. In fact, people who obsessively search for information related to disasters will demonstrate both types of processing. For example, after an earthquake or wildfire, people in at-risk regions may quickly accept safety advice from authorities without further investigation. If the issue is important enough, they may unquestioningly accept the information they receive about the disasters and will not look for more information, or sometimes, they will seek to judge the information received about the disasters or judge it with caution. Studies such as Johnson^44^ and Zhu et al.^30^ have reinforced the concept that information processing is prejudiced by information seeking.

Findings indicated that source credibility did not, however, significantly affect systematic processing and heuristic processing. The study of Ryu and Kim^31^ also confirmed the positive effect of source credibility on heuristic processing, although its impact on systematic processing was insignificant. In contrast, Zhang et al.^33^ showed that source credibility positively affected the quality of argument and systematic processing.

According to the findings, insufficiency of information did not have a significant effect on systematic and heuristic processing, although previous studies^30,44^ emphasize that insufficient information is a driver for systematic strategies in data processing.

Heuristic and systematic processing affected information credibility, which supports prior studies^29,34,51^. Therefore, whether people process information based on arguments or cues, the perceived information credibility will be more. For instance, during the COVID-19 pandemic, people who quickly accepted health guidelines from trusted sources like the WHO (heuristic processing) without questioning the details often perceived the information as credible and were less likely to fact-check.

Heuristic and systematic processing have directly influenced the intention to fact-check. People who rely more on arguments and cues after receiving news are more skeptical about the credibility of information and tend to use fact-checking tools. Systematic and heuristic processing have influenced people’s intention to fact-check indirectly by influencing information credibility. According to our findings, information credibility has had a direct negative influence on the intention to fact-check. In fact, people who think that social media convey complete, correct, and necessary information that is helpful in disaster-risk reduction and have perceived high credibility, had less intention to fact-check. As we expect, people with higher heuristic processing and checking perceived information have less credibility and had a greater intention to use fact-checking tools. These findings support previous studies^30,55^.

### Theoretical implications

Although dual-process theories are increasingly being used to explain information processing and examine information credibility at the individual level, no research has been found that examines the potential relevance of the heuristic–systematic model in understanding the predictors of users’ decision to use fact-checking tools on social media. The present study extends previous studies that examine factors associated with two routes (i.e., central and peripheral routes) or two modes (i.e., systematic and heuristic mode) of information processing (e.g., Zhu et al.^30^). In this study of the effects of systematic processing, heuristic cues (e.g., expertise, and source credibility) were strongly conducive to the intention to use fact-checking tools.

Our study contributes to the understanding of information-seeking as a key factor influencing both systematic and heuristic processing. We demonstrate that individuals who actively seek information, particularly in disaster contexts, are likely to engage in both types of processing. Depending on the perceived importance of the issue, they may either accept the information they encounter without further scrutiny or carefully evaluate it before forming judgments. By emphasizing this connection, our study underscores the importance of encouraging proactive information-seeking to enhance decision-making in disaster scenarios.

### Practical implications

We believe that this research will encourage journalists, social media users, and planners to use fact-checking tools to manage crisis situations.

First, fact-checking will give journalists greater certitude and confidence when putting out information during disaster situations. On social media advice on risk-reducing behavior will be taken more seriously, unsafe advice will be removed from the information cycle, and effective information will be dispensed more quickly. Without the challenge of rumors and false information to contend with, managers and planners will be able to focus more quickly and effectively on implementing risk reduction policies and helping those affected by disasters. Therefore, it is suggested that policymakers and planners facilitate information-seeking pathways to stimulate information processing. Although people will automatically form an opinion about the validity of information after conducting systematic or heuristic processing, the perceptions they form will lead them to programs that can more objectively detect fake information.

Systematic processing demands effort, which people under stress in disaster situations prefer to minimize; it is thus necessary for the arguments and necessary information for data analysis to be provided to the respondents in a simpler way.

In addition, information sources need to be improved, given that the findings will also have implications for policymakers and post-crisis management communicators. European countries can try to improve information transparency after crises. For example, a government could create a mechanism for information disclosure, namely regularly disseminate information to the public about the trends in deaths due to COVID-19 or through frequent training to provide the requisite information about disease control drugs.

## Conclusion and limitations

This study makes a significant contribution to the theoretical understanding of the use of HSM to predict intention to use fact-checking tools and also to investigate the impact of information processing and information credibility. We believe that the extended HSM provides an ideal theoretical framework for examining the psychological mechanisms underlying the intention to use fact-checking tools; it can thus contribute to the existing literature and be a first step toward predicting the use of fact-checking tools.

We tested HSM to examine perceived information credibility and then hypothesized and tested the intention to use fact-checking tools because of perceived information credibility and systematic and heuristic processing. This study shows that HSM provides a theoretical framework for future research in which quantitative and qualitative data are collected to further examine and test the research model and hypotheses.

This study also has limitations. Although the sample of respondents was selected from different European countries, it may not fully represent the broader population of the region. Additionally, the sample size of 202 respondents is relatively small for conducting SEM analysis. A larger sample would enhance statistical power and provide more robust and generalizable results. We suggest that future studies include larger and more diverse samples to address these limitations. Since the questionnaire was designed in English and conducted online, this may have restricted the representativeness of the sample by potentially excluding non-English speakers or those less likely to participate in online surveys, thereby introducing biases and limiting the diversity of perspectives in the analysis. We suggest that future studies consider using multilingual questionnaires and employing both online and offline survey methods to enhance inclusivity and representativeness.

The study was also published through e-mail and announcements on reliable and popular sites. Responders thus probably had a higher level of literacy than the norm.

Another limitation of this study is its focus on social network users in general. Past studies have emphasized that journalists need to predict breaking news and confirm the truth or untruth of social media content^56^. Future studies should thus consider journalists or politicians and planners in addition to social media general users.

While the reported fit indices (CFI, NFI, IFI, GFI, TLI) indicate a good overall fit for the structural equation model, the RMSEA value of 0.058, though within the acceptable range, suggests that the model could benefit from further refinement to achieve a closer fit to the data. Future studies with larger sample sizes and additional model testing may help improve the precision and overall fitness of the model.

The HSM was able to predict only 33% of the changes in the intention to fact-check. It is likely that many other factors, such as digital literacy and trust in fact checking websites, contribute to individuals’ intention to use fact-checking tools. These factors would thus merit attention in future studies.

## Data Availability

The data that support the findings of this study are available from the corresponding author, upon reasonable request.
